# Resolution of extravascular hemolysis with oral iptacopan monotherapy in a patient with treatment experienced paroxysmal nocturnal hemoglobinuria (PNH)

**DOI:** 10.1007/s00508-024-02390-w

**Published:** 2024-07-01

**Authors:** Wolfgang Füreder, Renate Thalhammer, Peter Valent

**Affiliations:** 1 Department of Internal Medicine I, Division of Hematology & Hemostaseology, Medical University of Vienna, Vienna, Austria; 2 Ludwig Boltzmann Institute for Hematology and Oncology, Medical University of Vienna, Vienna, Austria; 3 Department of Laboratory Medicine, Medical University of Vienna, Vienna, Austria

**Keywords:** PNH, Hemolysis, Complement, C5 inhibitors, Ferritin

## Abstract

Paroxysmal nocturnal hemoglobinuria (PNH) is an acquired hematologic disorder characterized by a loss of glycosyl-phosphatidyl-inositol-linked (GPI) proteins on various hematopoietic cells. Some GPI proteins are involved in the regulation of the complement system, and their absence renders erythrocytes susceptible to complement-mediated lysis. Current standard of care in PNH is to block the complement system at the level of C5 using ravulizumab or eculizumab; however, some patients with PNH may develop extravascular hemolysis (EVH) during treatment with C5 inhibitors. The proximal complement inhibitor iptacopan has recently been shown to be efficacious in patients with PNH. This article reports on a 43-year-old female patient with PNH who was successfully treated with iptacopan. The patient had received ravulizumab for several years and developed a clinically relevant EVH. After obtaining informed consent, the patient received oral iptacopan 200 mg twice daily and ravulizumab was discontinued. Over the next few weeks hemoglobin levels and reticulocyte counts normalized. The patient reported mild flushes with erythema, chills, and mild muscle pain, all of which resolved during follow-up. No breakthrough hemolysis occurred, and no severe adverse events were recorded.

## Introduction

Paroxysmal nocturnal hemoglobinuria (PNH) is a rare clonal hematologic disorder caused by an acquired deficiency of complement-regulating proteins on hematopoietic cells. In addition to hemolytic anemia, PNH is typically associated with thrombophilia [1].

The complement C5 inhibitor eculizumab has revolutionized treatment of PNH [2]. Currently the long-acting ravulizumab is the standard of care for PNH patients [3]; however, while intravascular hemolysis is suppressed by C5 inhibitors, some patients remain anemic while treated with these drugs. This may be attributed to extravascular hemolysis (EVH) related to deposition of C3 fragments on erythrocytes and subsequent clearance of these cells [4, 5]. Thus, anemia and thrombophilia are usually well-controlled by C5 inhibition, but in some patients, transfusion-dependent anemia persists [4, 5].

Proximal complement inhibitors targeting C3, factor B, or factor D may overcome EVH in affected PNH patients [6–8] as these agents have a greater potential to spare GPI- deficient erythrocytes from complement-mediated destruction compared to C5 inhibitors. As a result, the percentage of clonal erythrocytes (clone size) increases in these patients, and due to a larger size of GPI-deficient erythrocyte clones, breakthrough hemolysis (BTH) may be more serious than in patients treated with C5 inhibitors [5].

Iptacopan inhibits factor B in the alternative pathway of the complement system. In recent clinical trials, this drug has shown the potential to control extravascular and intravascular hemolysis in patients with PNH [8].

## Case reports

A 43-year-old female patient was diagnosed with PNH in 2006. She received ravulizumab in 2017. From November 2019 until June 2021 she participated in trials investigating BXC9930. After trial discontinuation, the patient received again ravulizumab 3300 mg every 8 weeks. Apart from PNH-targeting drugs, the patient has been treated with cabergoline for prolactinoma since 2006. The patient remained anemic despite ravulizumab therapy and also developed transfusion dependence.

Anemia, together with high reticulocyte counts, lactate dehydrogenase (LDH) < 1.5 × upper limit of normal (ULN, 250 U/L) and a Coombs test positive for C3d suggested EVH.

Pegcetacoplan, even though approved in Austria, was not refunded at our institution and therefore could not be used to treat EVH in our patient.

Iptacopan was supplied by Novartis within the framework of a Named Patient/Managed Access Program. After informed consent, the patient received iptacopan 200 mg orally twice daily beginning on day 41 after the last dose of ravulizumab. The patient had vaccine protection against infections with *Neisseria meningitidis, Streptococcus pneumoniae* and *Haemophilus influenzae*.

**Fig. 1:**
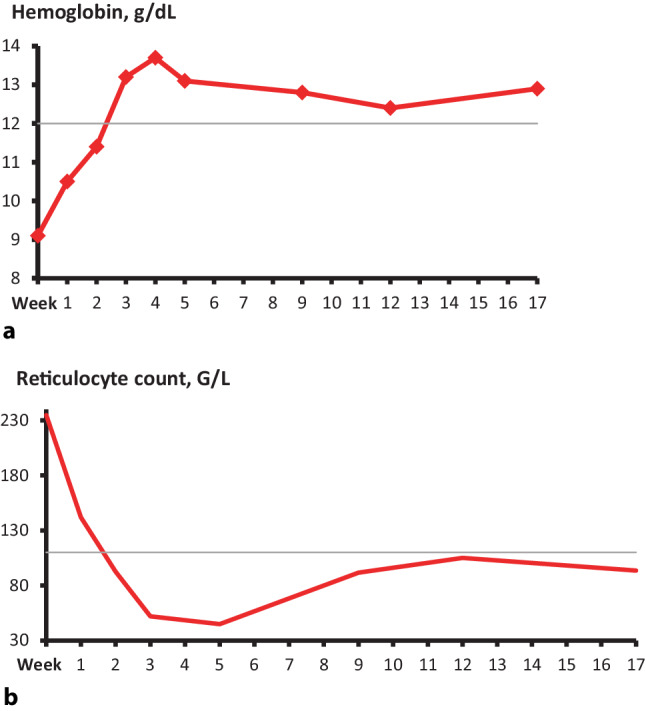
**a** Development of hemoglobin levels after the start of oral iptacopan. Following the initiation of iptacopan, the hemoglobin level increased rapidly from baseline and reached the normal range in week 3. Even though decreasing after a peak in week 4, hemoglobin remained within the normal range for the duration of follow-up. The *thin grey line* indicates the lower level of normal. **b** Development of reticulocyte counts after the start of oral iptacopan. Following the initiation of iptacopan, the reticulocyte counts decreased rapidly from baseline and reached the normal range in week 2. Even though increasing after a nadir in week 5, reticulocyte counts remained within the normal range for the duration of follow-up. The *thin grey line* indicates the upper level of normal

Following the start of iptacopan, the hemoglobin increased from 9.1 g/dL to normal range (NR, 12.0–16.0 g/dL) at week 3 and remained within NR during follow-up (Fig. 1a; Table 1). The reticulocyte count decreased from 234.8 G/L at baseline to NR within 2 weeks (NR 32–110 G/L) (Fig. 1b; Table 1). Haptoglobin remained below the detection limit. The FACIT fatigue score showed results comparable to healthy subjects at baseline and during follow-up (Table 1). The erythrocyte clone size was equivalent to the granulocyte clone size and remained stable at 96–100% during follow-up (Table 1).

**Table 1:** Patient characteristics at baseline and at weeks 1 to 17 after the start of iptacopan therapy

	BL	Week 1	Week 2	Week 3	Week 4	Week 5	Week 9	Week 12	Week 17
Hb g/dL	9.1	10.5	11.4	13.2	13.7	13.1	12.8	12.4	12.9
MCV, fL	117.3	113.9	110.5	105.9	103.1	102.0	98.7	101.8	101.8
Reti, G/L	234.8	141.9	92.5	52.0	nd	44.9	91.7	105.1	93.6
PLT, G/L	222	212	213	204	217	205	215	214	209
WBC, G/L	3.71	3.19	4.6	5.23	6.13	4.07	4.59	5.19	5.20
FACIT	46	49	50	49	nd	46	nd	52	48
Ery-clone, %	97	97	97	97	nd	97	96	100	96
Gran-clone, %	97	97	98	98	nd	98	98	98	98
Ferritin, μg/L	728.2	454.7	397.9	390.9	352.2	411.5	534.2	438.8	457.9
Transf, %	21.7	14.1	11.4	14.1	nd	22.5	18.3	15.7	15.7
Bili, mg/dL	0.61	0.29	0.38	0.38	0.44	0.42	0.52	0.48	0.44
LDH, U/L	296	198	177	190	210	214	297	276	251
Hapto, mg/dL	< 12	< 12	< 12	< 12	nd	< 12	nd	< 12	< 12

*BL* baseline, *Hb* hemoglobin, *MCV* mean corpuscular volume, *Reti* reticulocytes, *PLT* platelets, *WBC* white blood cells, *FACIT* FACIT fatigue score, *Ery-clone* erythrocyte clone size, *Gran-clone* granulocyte clone size, *Transf* transferrin saturation, *Bili* bilirubin, *LDH* lactate dehydrogenase, *Hapto* haptoglobin, *nd* not done

Due to red cell transfusions, the patient had developed moderate iron overload. The ferritin level amounted to 728.2 μg/mL before iptacopan therapy was started. Within 1 week of iptacopan, ferritin levels substantially decreased to 454.7 μg/L (Table 1).

After a peak in week 4, hemoglobin levels decreased slightly, and reticulocyte counts and LDH levels slightly increased, most probably indicating mild hemolysis (Fig. 1; Table 1).

Adverse events included chills without fever, mild muscle pain and minor flush symptoms with erythema, all of which subsided during follow-up.

After 17 weeks of iptacopan treatment, hemoglobin remained within NR and the FACIT fatigue score was stable. The reticulocyte counts, even though increasing from the trough values achieved, stayed within NR. No BTH or infections occurred.

## Discussion

Persistent anemia remains a challenge in some PNH patients treated with C5 inhibitors. Recently, drugs suppressing the complement system upstream of C5, such as pegcetacoplan, iptacopan and danicopan have been developed [6–8]. Pegcetacoplan is a C3 inhibitor that is administered as subcutaneous infusion twice a week [6]. Iptacopan, inhibiting factor B, is given orally twice daily [8]. Both of these drugs are used as monotherapy, whereas danicopan (given orally three times a day) targeting factor D is used as an add-on to C5 inhibition with eculizumab or ravulizumab [7]. Direct comparisons of these proximal complement inhibitors are lacking and algorithms to determine which drug best suits the requirements of specific patients need to be developed. This article reports on a PNH patient with EVH who was successfully treated with iptacopan monotherapy. In a clinical trial switching patients from C5 inhibition to iptacopan monotherapy, the drug showed superiority to C5 inhibition with increased hemoglobin levels, reduced fatigue and reticulocyte counts, and resulted in mean LDH levels < 1.5 × ULN [8].

Severe BTH is rarely seen in PNH patients receiving iptacopan [8]. In this patient mild hemolysis was identified in week 9 after starting iptacopan; however, hemolysis did not meet established criteria for BTH (with an increase of LDH > 2 × ULN) [3]. An explanation for this could be that the complement system “learned” how to escape blockage of factor B. Another possibility for mild hemolysis might be a compliance issue; however, this seems unlikely as the patient experienced a substantial benefit from therapy with iptacopan. In clinical trials with iptacopan mean hemoglobin levels from baseline to week 24 showed a mild decrease at week 12 [8]; however, interpretation of individual patient responses is limited.

Interestingly the patient had iron overload before iptacopan therapy and showed a drop in ferritin shortly after the start of iptacopan. An explanation for this finding might be an increased iron incorporation into a larger number of red cells. Once hemoglobin levels had stabilized, ferritin levels also stabilized, albeit now on a lower level. This has recently also been observed in a patient treated with danicopan^1^.

Intriguingly, haptoglobin remained below the detection limit under therapy despite a good clinical response to iptacopan. This observation may be explained by very mild hemolysis without anemia.

Adverse events in this patient included chills, flush and mild muscle pain. Whether these adverse events were indeed related to iptacopan remains unknown. All these events were not severe, resolved during follow-up and did not result in treatment discontinuation.

Together, iptacopan monotherapy has the potential to normalize hemoglobin levels and reticulocyte counts in PNH patients with EVH. In this patient, iptacopan was also well-tolerated without BTH or other relevant side effects.

## Footnotes

1 unpublished observation
